# Cases Where the Use of Oxpara Is Indicated

**Published:** 1904-04

**Authors:** E. F. Hazell

**Affiliations:** Springfield, Ill.


### CASES WHERE THE USE OF OXPARA IS INDICATED.

The caring for and curing of abscessed teeth has been more or less difficult to all practitioners, and will no doubt continue to cause unknown trouble in years to come.

This condition can be presented in two forms, the acute and the chronic. As you all know, when the case is presented in its early or acute stage, it can be more readily handled than when it has been allowed to be putrescent for some months, and has assumed the chronic form.

Our patients say, "I thought when a nerve was dead, it could not ache; how is it that this causes so much trouble?" It is this putrescent pulp which is the prolific source of so much annoyance to our patients.

To care for this condition without making it worse than when presented to us, has tested the skill of more than one man. We must open the tooth in question, remove the putrescent pulp and dress the case without forcing more of the septic matter through the foramen than has previously passed through.

In all of these cases, where it is at all possible, the rubber dam should be applied before anything else is done. Now wash the cavity with warm water containing some antiseptic solution, then remove all debris from the cavity, being careful not to produce pressure on the contents of the pulp canal. After this step, it has been found good practice to flood the cavity with a solution of hydrogen-peroxide ("Dioxogen"). Allow it to remain for a few minutes, so that at least a partial sterilization of the putrescent mass can take place. Then with a clean broach the contents of the pulp canal should be removed. This part of the operation must be done very promptly, and with the aid of a liberal quantity of peroxide. The cavity and canal should now be dried as thoroughly as possible, either with a blast-air or warm air syringe, or a compressed-air canal-drier. Many prefer the "Evans" dryer, but with this instrument there is danger of forcing some foreign matter through the foramen into the apical space.

The Oxpara powder and liquid, which has been previously mixed into a creamy consistency, is now to be used. If it is intended to fill permanently at this sitting, this paste is worked into the canal by means of a smooth broach, and then followed by a gutta-percha canal point. Should a permanent filling be not indicated, this preparation can be worked into the canal on a small piece of cotton, and allowed to remain for a few days, or as many weeks, when it should be cared for permanently. Some of the worst cases have yielded in from one to three treatments. A little pain is often felt after the insertion of this treatment, but this usually subsides in from twenty to thirty minutes.

Oxpara is a powder and liquid and is combined when ready for use. It is composed of the following drugs: formaldehyde, thymol, dried or burnt alum, and creosote. Dr. Dodez, the originator of the formula, worked for more than a year before he succeeded in getting the proportions just right, and then he tested this preparation for more than two years, before he placed it on the market.

It was with some misgivings that the writer tried this preparation, for so many so called "sure cure" preparations had been used with questionable results. Thus far, experience has proven oxpara entirely satisfactory, and it can be used with perfect safety, and always with the best results.—*Dr. E. F. Hazell, Springfield, Ill., in The Dental Summary.*

